# Medical activated charcoal tablets as a cheap tool for passive monitoring of gaseous ^131^I activity in air of nuclear medicine departments

**DOI:** 10.1007/s10967-018-6107-7

**Published:** 2018-08-14

**Authors:** Tomasz Mróz, Kamil Brudecki, Jerzy W. Mietelski, Mirosław Bartyzel, Ryszard Misiak, Andrzej Kornas

**Affiliations:** 1 Institute of Physics, Jagiellonian University, Łojasiewicza 11 st., 30-348 Cracow, Poland; 2 The Henryk Niewodniczanski Institute of Nuclear Physics Polish Academy of Sciences, Radzikowskiego 152 st., 31-342 Cracow, Poland; 3 Institute of Biology, Pedagogical University of Cracow, Podchorazych 2 st., 30-084 Cracow, Poland

**Keywords:** Passive monitoring, Nuclear medicine, Radioiodine, Gamma-ray spectrometry

## Abstract

It is well known that monitoring of radioactivity released from nuclear medicine departments is necessary to ensure the radiological safety of patients and personnel. Unfortunately, equipment for air sampling is often expensive, loud and is not suitable to use in hospitals. Our goal was to find cheap and simple system for passive monitoring of ^131^I activity concentration in the air of nuclear medicine departments. Medical activated charcoal tablets were used, because charcoal is excellent material for ^131^I trapping and tablets are readily available. Our proposed sampling protocol contains tablets preparation, exposure and measurements using HPGe detector. Different methods of tablets preparation (drying, impregnation with KI or NaOH) were tested while an experimental chamber was prepared for estimating ^131^I (released from Na^131^I, similar to that used in therapy) trapping efficiency of tablets in different conditions. Finally, tablets were placed in plastic holders and tested in nuclear medicine facilities.

## Introduction

In modern nuclear medicine, ^131^I is still one of the most widely used radionuclides as a short-lived (*T*_1/2_ = 8.03 d, where it’s effective half-life time in organisms is nearly equivalent to the physical half-live [1]) beta emitter for therapy and diagnostics of the thyroid gland. It can be used in many chemical forms, of which Na^131^I is the most popular, but other compounds such as ^131^I-metaiodobenzoguanidine and ^131^I-norcholesterol are also used [2]. Because activities of ^131^I used in medicine are very high (up to several dozen GBq), there is an enhanced risk of releasing ^131^I into the air and also to septic tanks systems [3]. There are several ways wich ^131^I can be released, e. g. opening packages with Na^131^I (used as a standard chemical form of ^131^I wich can be administrated for patients) or emitting gaseous ^131^I by patient (by breathing or perspiration). The gaseous fraction can be inhaled, and ^131^I can be deposited in thyroids of medical staff (technicians, nurses and doctors). ^131^I has been detected in medical staff using whole body spectrometer (WBS) and HPGe detectors. Activities of ^131^I measured in WBS were in the range from 5 ± 2 to 217 ± 56 Bq corresponding annual thyroid equivalent dose ranging from 0.4 mSv (for a female nurse) to 15.5 mSv (for a male technician) [4]. Due to these results, measurements of airborne ^131^I activity were performed at the Department of Endocrynology and Nuclear Medicine Holycross Cancer Centre (E&M HCC Kielce, Poland) where the previously measured medical staff is worked. Results showed, that the activity of ^131^I in the work space ranged from 28 ± 1 to 492 ± 4 Bq m^−3^ depending on location (nurses station or hot room) which was similar to results obtained by other authors [2, 5]. With this levels of ^131^I concentration in the workplace the annual inhalation effective dose can range from 0.47 mSv (female nurse) to 1.3 mSv (male technician) [6]. ^131^I activity was measured with a charcoal trap covered with a Petryanov filter cloth and was connected to a mobile HVS 30 aerosol sampler. This combination, allowed us to measure both, aerosols (trapped on filter) and the gaseous fraction of ^131^I with high efficiency. Unfortunately, this equipment is not a good choice to be routinely used in hospitals due to the size and noise of the unit. Therefore, we developed a passive ^131^I monitoring technique, similar to ^222^Rn monitoring with a PicoRad system. In order to introduce a cheap and simple system, charcoal tablets, which are readily available and are commonly used in medicine, were used. Because charcoals are often impregnated, (e.g. with KI or Ag salts) some modification of tablets to compare them with non-treated tablets were performed. This study is similar to that presented by Jimènez et al. [2] but the activity of ^131^I is measured directly, without the chemical treatment, that is necessary for LSC measurements. It is a practical approach, because nuclear medicine departments are equipped with counting systems, and therefore facilities can make measurements independently.

## Materials and methods

### Charcoal tablets preparation

During the in situ experiment three methods of tablet preparation for ^131^I trapping were tested. First, tablets were impregnated in 2% solution of KI in water. The second was impregnation of tablets with 2% NaOH solution. Both the KI and NaOH tablets were baked at 105 °C and impregnated by dropping of solution onto surface of the tablets using a plastic Pasteur pipette. After impregnation, tablets were held at room temperature by one hour and then were baked again for 24 h at 105 °C to remove water. The third method of tablet preparation used only baking, without any wet impregnation.

### Containers

Empty PicoRad containers were filled with dry silica gel to reduce moisture exposure of tablets during transport and to stabilize moisture variations during sampling. We have used silica gel with a color indicator, which slowly becomes greenish during exposure of the tablets at the hospital, indicates that the silica gel still had some moisture capacity. Dry charcoal tablets were placed into small plastic holders with Petryanov filter cloth on the top and on the bottom side. For each PicoRad container we have placed two tablets. Then, containers were closed, sealed with Parafilm and sent to the hospital for ^131^I collection.

### Sampling and activity measurement

Patient rooms and hot rooms were chosen as a sampling sites. In both sites we placed a set of three containers and left them open for 1 week. After exposure, containers were sent back to our laboratory for gamma-ray spectrometry measurements, performed using HPGe detector with 9% relative efficiency constructed at the Institute of Nuclear Physics Polish Academy of Sciences. Separate measurements of the tablets, silica gel and filter cloths were performed.

### Preparation of calibration chamber

The sampling system was also tested using a calibration chamber and a system for producing tracer isotopes. Production of tracer (^126^I) is possible by irradiation of non-enriched TeO_2_ with a proton beam at 30 MeV (corresponding to the highest cross-section for ^126^I [7]), using AIC-144 cyclotron (Institute of Nuclear Physics PAS). Activated targets are heated in the furnace up to 750 °C and gaseous iodine is trapped in 0.01 N NaOH solution. From the basic solution, iodine can be released in gaseous ^126^I_2_ by adding concentrated HNO_3_. The test chamber was prepared from a glovebox sealed with chemical-resistant silicone and equipped with a Geiger based counting system for detecting eventual leaks form the chamber.

## Results and discussion

Results of the gamma spectrometry measurements are presented in Table 1. There was no ^131^I detected on silicagel or the filter cloths. The mean trapping efficiency calculated by the simple activity ratio method ranges from 0.13 to 2.9%.

**Table 1:** ^131^I activities measured in charcoal tablets after exposure

Sampling location	Tablets type	Gaseous ^131^I activity (Bq)
Hot room	Baked only	4.71 ± 0.82
	2% KI and baked	3.33 ± 0.74
	2% NaOH and baked	0.83 ± 0.48
Nurses station	Baked only	0.59 ± 0.36
	2% KI and baked	0.19 ± 0.16
	2% NaOH and baked	0.23 ± 0.18

The highest activity in both sampling stations was measured in tablet, which were baked at 105 °C without any wet treatment. The total adsorption of ^131^I on activated charcoal with chemical modification is the sum of the adsorption process on charcoal itself and chemical trapping by the impregnating agent. In the case of KI and NaOH impregnation these processes can be described as follows:

1 $${\text{KI}} + {^{131} {\text{I}}_{2}} \to {\text{ K}}^{131} {\text{I}}_{3}$$

2 $$^{131} {\text{I}}_{2} + 2{\text{NaOH }} \to {\text{Na}}^{131} {\text{I }} + {\text{Na}}^{131} {\text{IO}} + {\text{H}}_{2} {\text{O}}$$

However, impregnation can cause a reduction of active surface and with higher concentrations of impregnating solution absorption will be reduced due to occupation of the active surface [8]. Also, the impregnation technique is likely important. In first approach we used an impregnation method described by Gourani et al. [9], but commercially made medical tablets degraded in impregnating solution. Later, we attempted to use Pasteur pipettes to gently, drop by drop, impregnate form both sides of a tablet. However, the results showed that “stock”, baked-only tablets should also be a good choice as a passive monitoring absorption material. Further studies will show if impregnation with a reduced concentration of impregnating solution is viable. Another issue in the case of passive monitoring is the calibration of the monitoring system, where it is the goal to find the relationship between activity absorbed in the activated charcoal and the activity concentration in air. In a first approximation for our pilot study we have calculated the trapping efficiency as a ratio between the activity trapped in tablets and the activity in the air of the sampling site as determined by conventional sampling with HVS-30 station. However, this approach is only a rough estimation, and tablets must be tested in controlled conditions. For future efficiency tests, we have decided to use ^126^I as a tracer, because it has a half-life almost two times longer than ^131^I (13.11 d) and is easy to produce on site with a cyclotron from non-enriched targets (TeO_2_) using the reaction ^nat^Te(*p,xn*)^124,126^I. Therefore transport of highly radioactive Na^131^I (commercially available) is not necessary. ^222^Rn trapping efficiency can also be used for calibration, where the standard equation used for calibration of activated charcoal detectors in a chamber is [2]:

3 $$C_{\text{a}} V = N/(f_{\text{a}} f_{\upgamma} t \, k)$$

where *C*_a_ (Bq m^−3^) is concentration inside glovebox during calibration, *V* (m^3^) is the chamber volume, *N* is the count rate of the calibrated sample (cps), *f*_a_ is the trapping efficiency (Bq m^−3^ cps^−1^), *f*_γ_ is the germanium spectrometer efficiency for the gamma line (364 keV for ^131^I or 666 keV for ^126^I), t is the charcoal exposure time in the chamber (s) and *k* is the correction factor for radioactive decay during calibration and counting (s^−1^) calculated as:

4 $$k = \lambda t/\left( {1 - e^{( - \lambda \, t)} } \right)$$

where *λ* is the decay constant of ^131^I or ^126^I (depending on the isotope used for calibration). The issue with this is that there may be only small amounts of charcoal used in a single container, which may only provide a weak signal in small HPGe detectors. However, the application of germanium detectors with high efficiency or with a well geometry should give quite good results.

## Conclusions

This article describes results of pilot studies about the application of medical activated charcoal for passive monitoring of gaseous ^131^I activity concentration in nuclear medicine departments, where activity concentration of ^131^I can reach levels of few hundred Bq. Conventional air sampling methods are based on large and noisy air samplers, which are not suitable for routine measurements in hospitals. Our method is based on direct gamma-ray measurements of ^131^I trapped in charcoal tablets prepared in different ways using a HPGe detector, requiring no chemical treatment or mixing with scintillating cocktail. Results showed, that ^131^I can be trapped in both, impregnated and non-impregnated tablets. Since measurements of ^131^I activity in air are necessary for the radioprotection of patients and personnel after precise calibration this method will become useful.
